# DS-1205b, a novel selective inhibitor of AXL kinase, blocks resistance to EGFR-tyrosine kinase inhibitors in a non-small cell lung cancer xenograft model

**DOI:** 10.18632/oncotarget.27114

**Published:** 2019-08-27

**Authors:** Takeshi Jimbo, Mana Hatanaka, Takahiro Komatsu, Tomoe Taira, Kentaro Kumazawa, Naoyuki Maeda, Takashi Suzuki, Masahiro Ota, Noriyasu Haginoya, Takeshi Isoyama, Kosaku Fujiwara

**Affiliations:** ^1^ Oncology Function, Daiichi Sankyo Co., Ltd., Tokyo, Japan; ^2^ Quality & Safety Management Division, Daiichi Sankyo Co., Ltd., Tokyo, Japan; ^3^ Biologics Division, Daiichi Sankyo Co., Ltd., Tokyo, Japan; ^4^ Research Management Department, Daiichi Sankyo RD Novare Co., Ltd., Tokyo, Japan; ^5^ Medical Affairs Division, Daiichi Sankyo Co., Ltd., Tokyo, Japan

**Keywords:** AXL, DS-1205, EGFR-TKI resistance, erlotinib, osimertinib

## Abstract

The AXL receptor tyrosine kinase is involved in signal transduction in malignant cells. Recent studies have shown that the AXL upregulation underlies epidermal growth factor receptor (EGFR)-tyrosine kinase inhibitor (TKI) resistance in EGFR-mutant non-small cell lung cancer (NSCLC). In this study, we investigated the effect of DS-1205b, a novel and selective inhibitor of AXL, on tumor growth and resistance to EGFR TKIs. In AXL-overexpressing NIH3T3 cells, DS-1205b potently inhibited hGAS6 ligand-induced migration *in vitro* and exerted significant antitumor activity *in vivo*. AXL was upregulated by long-term erlotinib or osimertinib treatment in HCC827 EGFR-mutant NSCLC cells, and DS-1205b treatment in combination with osimertinib or erlotinib effectively inhibited signaling downstream of EGFR in a cell-based assay. In an HCC827 EGFR-mutant NSCLC xenograft mouse model, combination treatment with DS-1205b and erlotinib significantly delayed the onset of tumor resistance compared to erlotinib monotherapy, and DS-1205b restored the antitumor activity of erlotinib in erlotinib-resistant tumors. DS-1205b also delayed the onset of resistance when used in combination with osimertinib in the model. These findings strongly suggest that DS-1205b can prolong the therapeutic benefit of EGFR TKIs in nonclinical as well as clinical settings.

## INTRODUCTION

AXL, a member of the mammalian TYRO3, AXL, and MER (TAM) receptor kinase family, is a cell-surface transmembrane receptor that exerts regulated kinase activity through its cytoplasmic domain. It plays important roles in migration, invasion, cell cycle, and drug sensitivity in malignant cells [1–3]. The transforming gene *AXL* (derived from the Greek word “anexelekto,” which means uncontrolled) was originally isolated from chronic myelogenous leukemia cells, is located on chromosome 19q13.2, and encodes 20 exons [4]. AXL is ubiquitously expressed in various organs and cells and is overexpressed in several human cancers, including lung [5–8], colon [9, 10], esophageal [11, 12], breast [13, 14], astrocytoma-glioblastoma [15], and hematological cancers [16–18]. AXL is therapeutic target in some cancers [8, 17, 19]. GAS6 is the major ligand for TAM receptor tyrosine kinases and, particularly, is the sole ligand for AXL [20]. The binding of GAS6 to its receptors promotes cancer cell proliferation, survival, and migration *in vitro* [1, 2, 21].

AXL expression is upregulated in certain tumors resistant to molecular targeting [16, 22–25] and chemotherapeutic drugs [25–28], and AXL siRNA- or shRNA-mediated knockdown improved the sensitivity of resistant cells. AXL and MER receptor tyrosine kinases reportedly play key roles in the resistance to multiple anticancer therapies [29]. Brand et al. identified AXL as a key mediator of cetuximab resistance, providing a rationale for the clinical evaluation of AXL-targeting drugs to treat cetuximab-resistant cancers [30]. AXL expression can bypass resistance to targeted agents and specifically, to inhibitors of other RTKs, by maintaining pathway activity via alternative effectors or by inducing the activation of other signaling networks [29]. Increased AXL expression has been correlated with resistance to both chemotherapeutic drugs and targeted agents [31].

Human non-small cell lung cancer (NSCLC), with activating mutations in epidermal growth factor receptor (EGFR), responds very well to treatment with EGFR-targeted tyrosine kinase inhibitors (TKIs), such as erlotinib and gefitinib; however, these responses are reduced by acquired resistance [32]. Several mechanisms underlie the acquired resistance, and among them, the amino acid alteration from threonine^790^ to methionine^790^ (T790M) in EGFR and amplification of MET as a secondary genetic alteration are found in more than 50% of resistant tumors [33]. In addition, recent studies have reported that approximately 20% of patients who develop resistance to erlotinib show enhanced AXL expression, suggesting that AXL-mediated signaling may be involved in acquired resistance [5, 7]. Furthermore, it has been recently reported that AXL confers intrinsic resistance to osimertinib, a third-generation EGFR-TKI, and accelerates the emergence of tolerant NSCLC cells [34]. Thus, AXL inhibition may prevent or overcome acquired resistance to EGFR TKIs [5].

In this study, we evaluated the effect of DS-1205b, a novel, specific, small-molecule inhibitor of AXL kinase, on tumor growth and resistance to EGFR TKIs. Our findings strongly suggest that combination treatment with DS-1205b can prolong the therapeutic benefit of EGFR TKIs. Phase I clinical trials of DS-1205c in combination with osimertinib or gefitinib are underway, and information on these studies can be found at https://clinicaltrials.gov/ (ID: NCT03255083; NCT03599518). Both DS-1205b and DS-1205c are sulfate hydrates, with similar stoichiometries.

## RESULTS

### DS-1205b is a potent and highly selective AXL inhibitor

**Figure 1:**
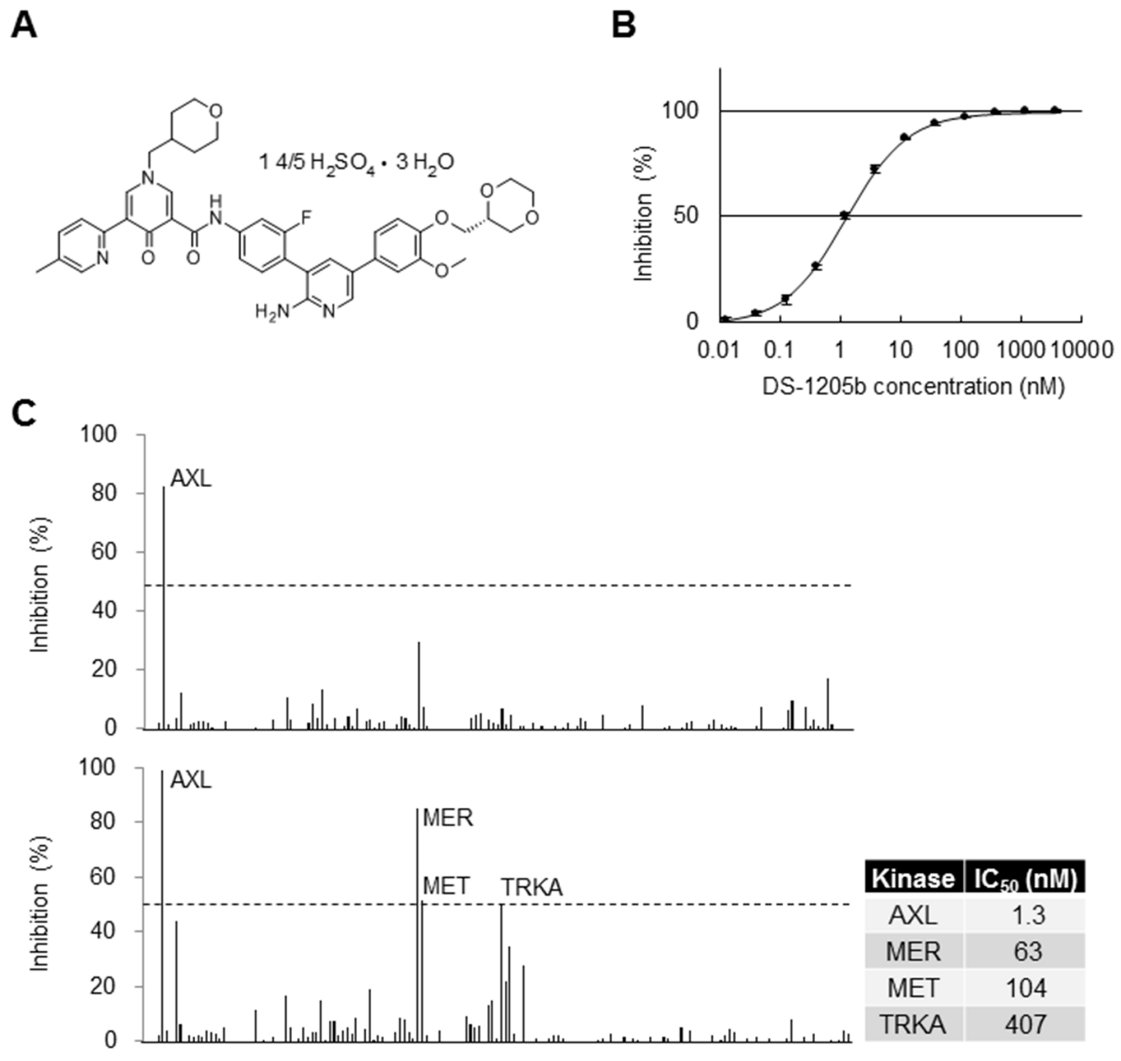
Selectivity of DS-1205b in biochemical assays. **(A)** Structural formula of DS-1205b. **(B)** Inhibition curve of DS-1205b against AXL kinase in a mobility shift assay using recombinant AXL protein. The graph shows mean inhibition at each concentration point with the SD (*N* = 4). **(C)** Kinase selectivity of DS-1205b was evaluated by mobility shift assay using 161 kinases (Supplementary Table 1), in the presence of 1 mM ATP. Upper panel: 13 nM (approximately IC_80_), lower panel: 200 nM. The IC_50_ values of DS-1205b toward AXL, MER, MET, and TRKA kinases were determined by mobility shift assay in the presence of 1 mM ATP using recombinant human AXL, MER, MET, and TRKA proteins.

The chemical structure of DS-1205b is shown in Figure 1A. DS-1205b is a novel compound discovered and synthesized by Daiichi Sankyo Co., Ltd. This report is the first disclosure of the compound structure; experimental details of synthesis are described in the Supplementary Methods.

The AXL inhibition curve of DS-1205b is shown in Figure 1B; the 50% inhibitory concentration (IC_50_) was 1.3 nM. The kinase selectivity of DS-1205b was examined for 161 kinases by mobility shift assay in the presence of 1 mM ATP, which is a near-physiological condition. None of the kinases were inhibited by more than 30% at 13 nM (the IC_80_ value for AXL which is potent inhibition concentration; upper panel in Figure 1C), and MER, MET, and TRKA kinases were inhibited by more than 50% at 200 nM DS-1205b (lower panel in Figure 1C). The IC_50_ values of MER, MET, and TRKA were 48-, 80-, and 313-fold that of AXL, and clear difference was confirmed. Thus, DS-1205b is a highly selective and potent AXL kinase inhibitor. DS-1205b showed potent and highly selective inhibitory activities toward AXL kinase.

### *In vitro* and *in vivo* activities of DS-1205b in NIH3T3-AXL cells

**Figure 2:**
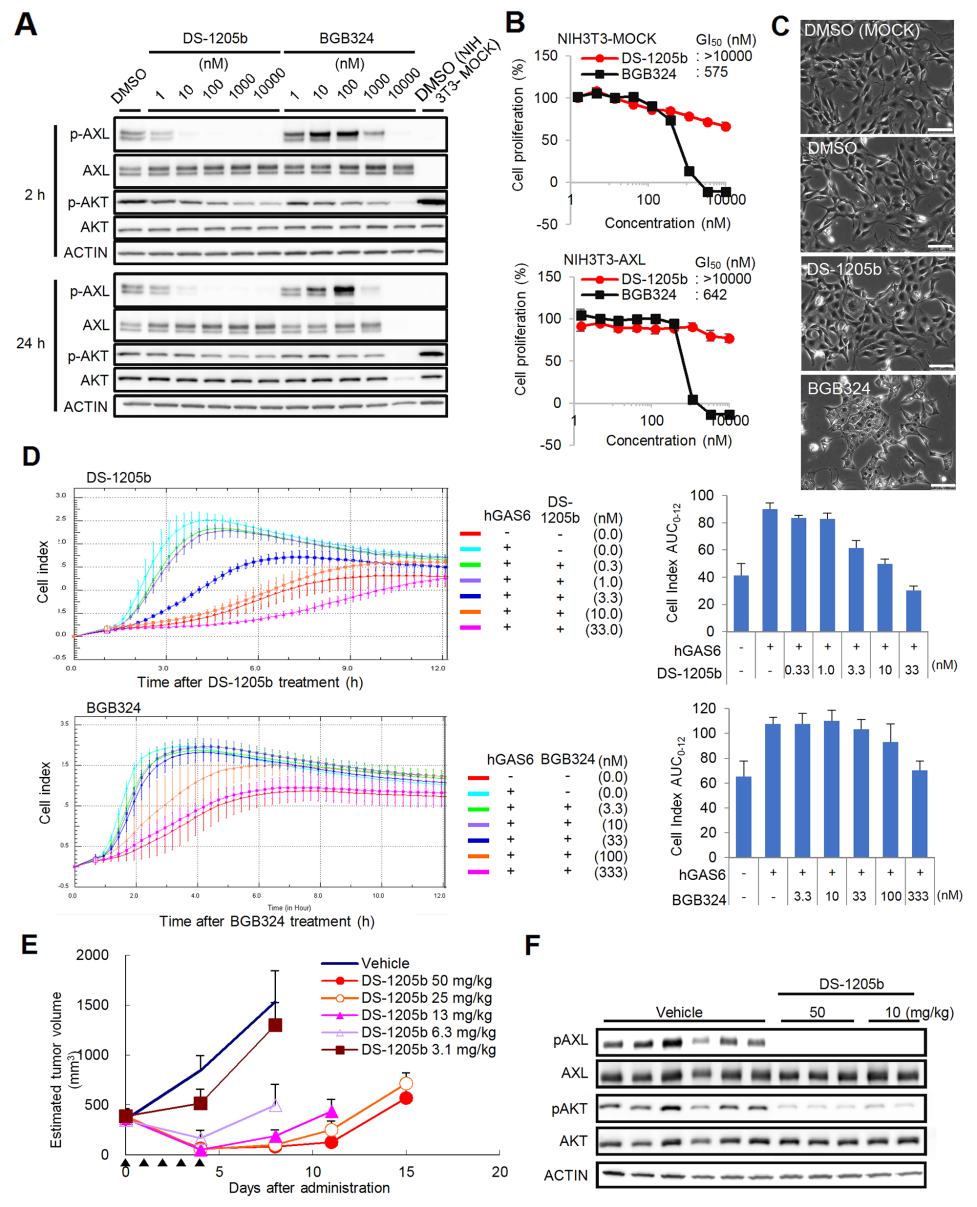
Effects of DS-1205b and/or BGB324 in NIH3T3-AXL cells *in vitro* and *in vivo*. **(A)** *In vitro* phosphorylation inhibition assay of AXL and AKT. NIH3T3-AXL cells were treated with DS-1205b or BGB324 for 2 h or 24 h and then subjected to western blot analysis. Detailed information on the antibodies used is given in the Materials and Methods. **(B)** Growth inhibitory activities as assessed by ATP assay. The graph shows mean inhibition at each concentration point with the SD (*N* = 3). **(C)** Photographs show cell morphological changes after 24 h of treatment with DMSO, 1 μM DS-1205b, and 1 μM BGB324, respectively. Scale bar represents 100 μm. **(D)** Inhibitory activities of DS-1205b or BGB324 on hGAS6-induced migration in NIH3T3-AXL cells. The cell index (CI; a dimensionless parameter) was derived as the relative change in measured electrical impedance to represent cell migration. hGAS6: 500 ng/mL. Bars indicate the SD (*N* = 4). The CI AUC_0–12_ indicates the sum of each CI for 12 h, and ΔAUC_0–12_ indicates the difference between the CI AUC_0–12_ of the DMSO-treated control group and the experimental group. **(E)** Effects on growth of NIH3T3-AXL cells *in vivo*. Data are the mean ± SE (*N* = 5; NOD-SCID). Arrowhead: administration day (po, bid). **(F)** *In vivo* phosphorylation inhibition assay of AXL and AKT. Tumor samples taken after 2 h of DS-1205b administration were subjected to western blot analysis. Detailed information on the antibodies used is given in the Materials and Methods.

To clarify the potency of DS-1205b as a selective AXL inhibitor, protein phosphorylation, growth, and migration were assessed in artificially AXL-overexpressing mouse embryonic fibroblast cells NIH3T3 (NIH3T3-AXL cells). BGB324, a forefront runner of AXL kinase inhibitors in clinical stage, was used for comparison [35]. Phosphorylation of AXL was completely inhibited upon 2 h treatment with DS-1205b at concentrations above 10 nM, whereas phosphorylation of AKT serine/threonine kinase, its downstream factor, was slightly inhibited, in a dose-dependent manner (Figure 2A). AXL phosphorylation was notably enhanced by BGB324 at 10–100 nM and inhibited at concentrations above 1,000 nM. After 24 h treatment with 10,000 nM BGB324, there were no signals for AXL and AKT, as most cells were killed. Cell proliferation decreased gradually upon DS-1205b treatment, but growth was not obviously inhibited (GI_50_: >10,000 nM; Figure 2B). However, the GI_50_ of BGB324 for AXL was 642 nM in NIH3T3-AXL cells, and cell viability was clearly affected at concentrations above 1,000 nM. The similar results were obtained when AXL ligand hGAS6 was added to examine AXL and the downstream signaling activation (Supplementary Figure 1). We also observed cell morphological changes upon drug treatments. Cytoplasmic vacuoles were observed within 24 h after treatment with 1,000 nM BGB324, whereas treatment with 1,000 nM DS-1205b induced no clear changes (Figure 2C). After 24 h, no viable cells were observed by microscopy (data not shown).

To evaluate the effects of DS-1205b and BGB324 on NIH3T3-AXL cell migration, a cell index (CI) was calculated as the relative change in measured electrical impedance to represent cell migration. DS-1205b significantly suppressed the CI at 3.3–33 nM, and the half maximal effective concentration (EC_50_) was 2.7 nM (Figure 2D). BGB324 significantly suppressed the CI at concentrations above 333 nM, and the EC_50_ was 132.3 nM.

Antitumor effects of DS-1205b were assessed in mice bearing subcutaneously implanted NIH3T3-AXL cells. Tumor growth was inhibited by 39–94% at doses of 3.1–50 mg/kg, and a statistically significant antitumor effect was observed at doses above 6.3 mg/kg (3.1 mg/kg: *P < 0.01*; 6.3–50 mg/kg: *P < 0.001* vs. vehicle-treated control by parametric Dunnett’s test using the data on day 4; Figure 2E). The dose-dependent antitumor effect of DS-1205b on day 4 was confirmed by Spearman’s rank correlation testing (*P* < 0.0001). In addition, DS-1205b induced tumor regression by 54–86% at doses of 6.3–50 mg/kg. We also examined the effect of DS-1205b on AXL and AKT phosphorylation in tumors. Phosphorylation of both proteins was clearly reduced, suggesting that the antitumor effect of DS-1205b monotherapy in this model is mediated by pAXL inhibition (Figure 2F).

### Generation of erlotinib- or osimertinib-resistant cells and AXL expression

**Figure 3:**
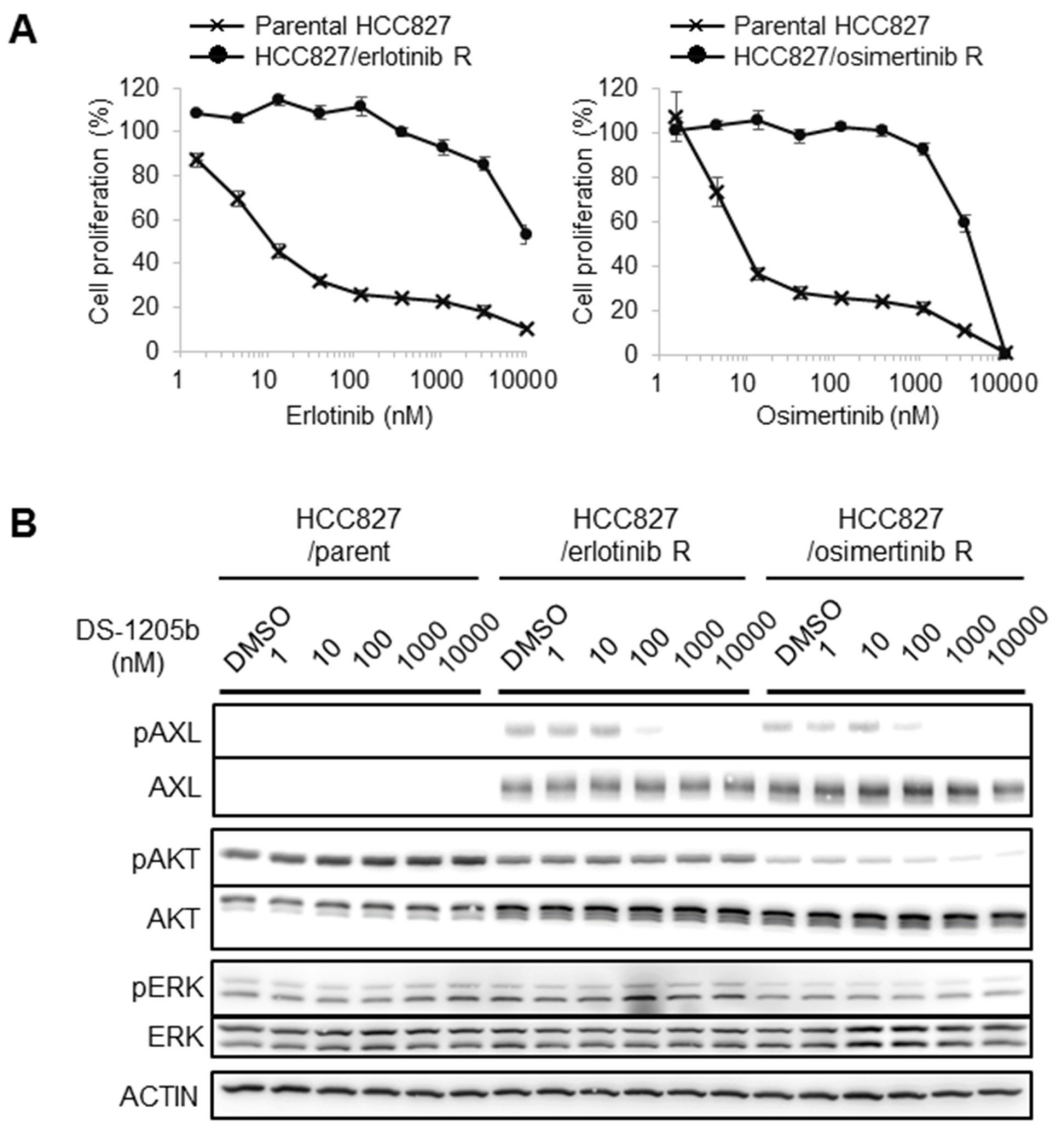
Generation of erlotinib- or osimertinib-resistant cells and AXL expression. **(A)** HCC827 cells were treated with erlotinib or osimertinib at 1,000 nM to generate acquired resistance. IC_50_ values were determined on day 80 after starting drug treatments. **(B)** Parental, erlotinib-, or osimertinib-resistant HCC827 cells were treated with 1, 10, 100, 1,000, or 10,000 nM DS-1205b for 2 h and subjected to western blot analysis on day 52 (for the erlotinib resistant cells) or day 54 (for the osimertinib resistant cells). Detailed information on the antibodies used is given in the Materials and Methods.

We generated HCC827 human NSCLC cells with acquired resistance to erlotinib or osimertinib by treating them for 80 days with 1,000 nM erlotinib or osimertinib, and we examined AXL expression by western blotting to evaluate the potential relation between acquired resistance and AXL upregulation. In parental HCC827 cells, the IC_50_ values of erlotinib and osimertinib were 11.3 nM and 9.2 nM, respectively, whereas in erlotinib- and osimertinib-resistant HCC827 cells, the IC_50_ values were 4,278.4 nM and 3,975.9 nM, respectively (Figure 3A). AXL expression was obviously upregulated in both erlotinib- and osimertinib-resistant HCC827 cells, and the phosphorylation was inhibited by DS-1205b treatment in a dose-dependent manner (Figure 3B). DS-1205b treatment tended to suppress AKT phosphorylation in osimertinib-resistant HCC827 cells. It is also important to examine the other putative molecular alterations present in these resistance cells. We had an additional experiment for understanding of potential other alterations using RTK array (Proteome Profiler Human Phospho-RTK Array Kit by R&D, #ARY001B). The reduction of phosphorylation levels of EGFR, ERBB2, ERBB3, MET, and RET, or the increase of INSR and IGF1R were observed in both resistant cells (Supplementary Figure 2). The possibility that dominant pathway on growth and survival of the NSCLC cells shifts from EGFR pathway partially to bypass signal pathways such as AXL by EGFR inhibitor treatment is considered, however, it is not clear.

### Combination treatment with erlotinib or osimertinib and DS-1205b inhibits AXL signaling *in vitro*

**Figure 4:**
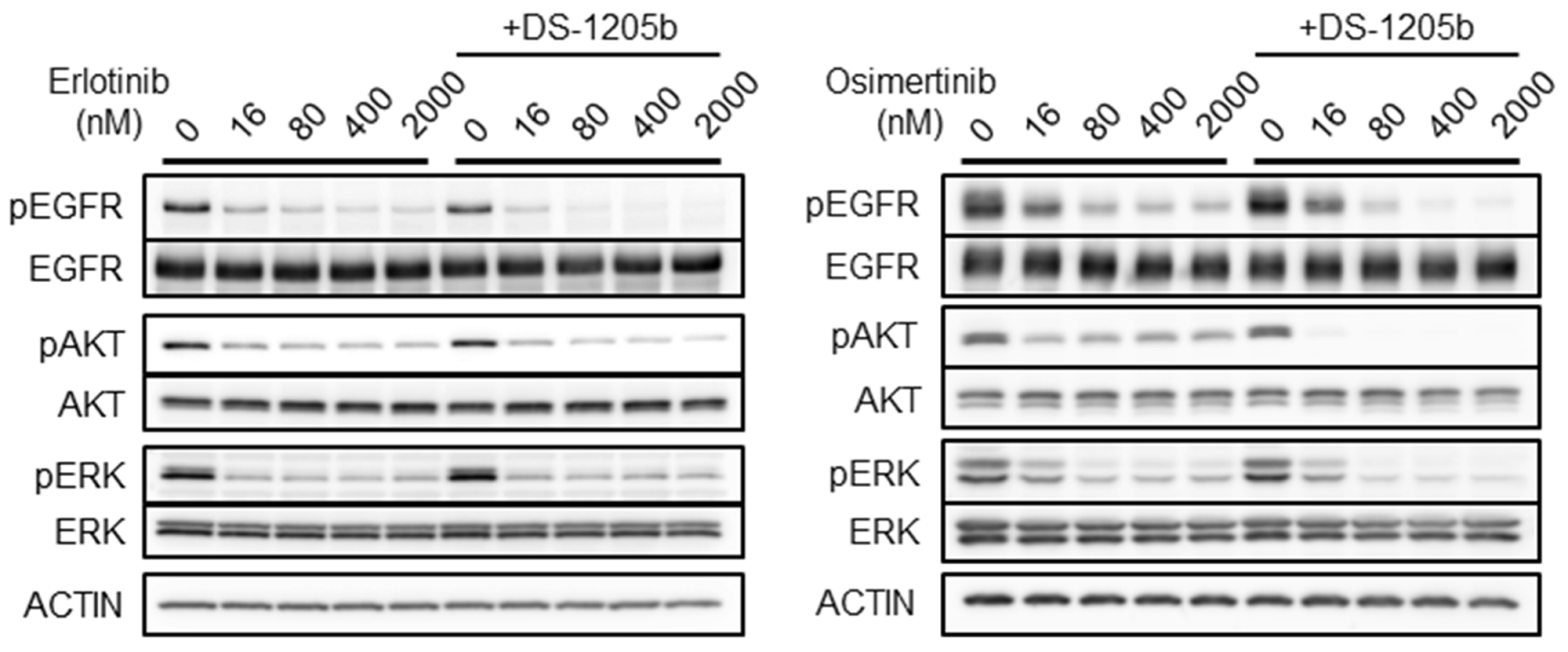
Combination treatment with erlotinib or osimertinib and DS-1205b inhibits AXL signaling *in vitro*. HCC827 cells were treated with erlotinib or osimertinib at 16, 80, 400, or 2,000 nM. In case of cotreatment, DS-1205b was used at 1 μM in this assay. After 2 h treatment, the HCC cells were collected and evaluated by western blot analysis. Detailed information on the antibodies used is given in the Materials and Methods.

We examined the effects of combined treatment with erlotinib or osimertinib and DS-1205b on EGFR and AXL downstream signaling with the aim to elucidate the mechanism of action of the compounds, using a cell-based assay. As shown in Figure 4, when HCC827 cells were treated with erlotinib or osimertinib at 16, 80, 400, or 2,000 nM, EGFR, AKT, and extracellular signal-regulated kinase (ERK) phosphorylation was reduced. However, erlotinib or osimertinib alone could not completely inhibit phosphorylation, even at 2,000 nM. Cotreatment with erlotinib or osimertinib and DS-1205b significantly inhibited phosphorylation compared to each monotreatment, especially the combination with osimertinib. In particular, AKT phosphorylation was completely inhibited at the lowest doses of osimertinib and DS-1205b (16 nM). The co-treatment seems to be efficient only in osimertinib resistant cells by inhibition of AKT pathway.

### Antitumor effects of DS-1205b in an acquired erlotinib resistance subcutaneous xenograft model

**Figure 5:**
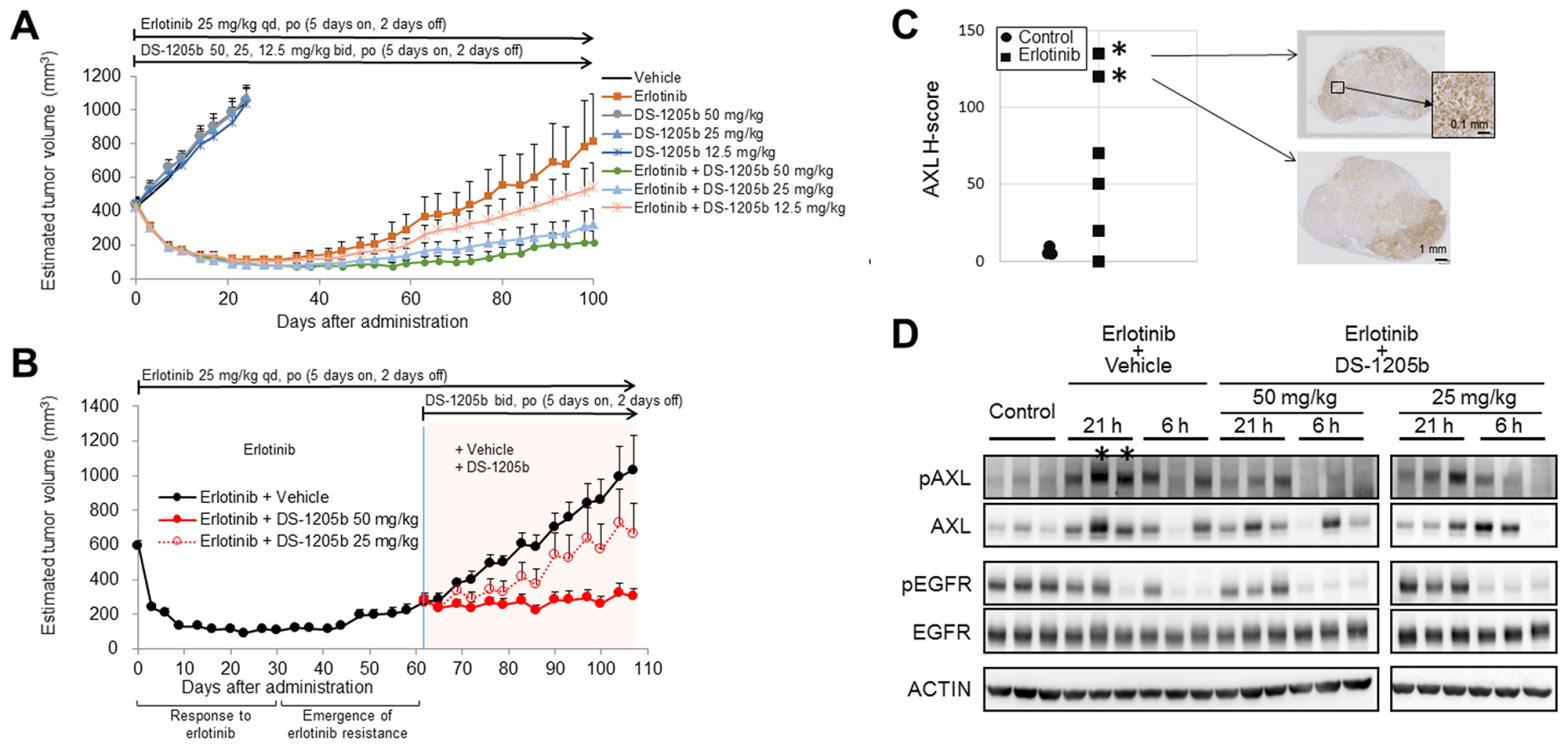
DS-1205b delays erlotinib acquired resistance and restores the treatment effect of erlotinib in an HCC827 sc xenograft model. **(A)** Addition of DS-1205b to erlotinib delays the onset of drug resistance in a human EGFR-mutant NSCLC xenograft model. Mice with subcutaneous xenograft human HCC827 tumors were treated as indicated. Data are the mean ± SE (*N* = 8; nude mice). **(B)** Antitumor activity of erlotinib was restored by addition of DS-1205b in an erlotinib acquired resistance xenograft model. Mice with subcutaneous xenograft human HCC827 tumors were treated as indicated. Data are the mean ± SE (*N* = 6). **(C)** AXL immunostaining images and AXL signal intensities and H scores for HCC827 xenograft tumors are shown. Immunohistochemistry of AXL is described in detail in the Materials and Methods. **(D)** AXL and EGFR expression was analyzed by western blotting in tumor samples obtained 24 days after tumor implantation for the control group or day 108 for the erlotinib-treated group in Figure 5B. “21 h” and “6 h” indicate the time in hours after the last treatment with DS-1205b. ^*^same tumor sources.

The antitumor effect of DS-1205b in combination with erlotinib was evaluated in nude mice subcutaneously inoculated with HCC827 cells. The mean tumor volumes on day 100 for groups administered erlotinib combined with DS-1205b at doses of 50, 25, or 12.5 mg/kg twice daily (bid) were 216.8, 321.0, and 541.7 mm^3^, respectively, whereas the mean tumor volume of the erlotinib monotherapy group (erlotinib: 25 mg/kg qd) was 814.5 mm^3^. Tumor volumes in the erlotinib plus high-dose DS-1205b group on days 59–80, 98, and 100 were significantly (*P* = 0.0257–0.0469 by parametric Dunnett’s test) lower than those in the erlotinib group (Figure 5A). As DS-1205b showed no antitumor effect, these results suggested that combined treatment with DS-1205b and erlotinib may delay the onset of acquired resistance to erlotinib.

Next, we examined the effect of DS-1205b plus erlotinib in HCC827 tumors *in vivo* after acquired resistance to erlotinib was established. Tumors started to regrow after 48 days of treatment with erlotinib, and DS-1205b treatment was initiated at day 62. DS-1205b inhibited tumor growth by 97% and 47% at doses of 50 and 25 mg/kg, respectively. A statistically significant antitumor effect was observed at each measurement point for DS-1205b at 50 mg/kg (*P* = 0.0100) (Figure 5B). DS-1205b restored the antitumor activity of erlotinib in a dose-dependent manner, which was confirmed by Spearman’s rank correlation coefficient testing (*P* = 0.0002). These results suggested that DS-1205b restores the antitumor activity of erlotinib in combination treatment.

Immunostaining for AXL expression and H scores for HCC827 xenograft tumors are shown in Figure 5C. The erlotinib-treated groups had higher H scores than the non-treated group (mean ± standard deviation (SD) for H score, 6.7 ± 2.9 for the non-treated group (control); 65.8 ± 53.7 for erlotinib monotherapy). AXL expression was heterogeneously distributed in the tumor cells, although AXL-expressing and non-AXL-expressing tumors were not morphologically distinct.

AXL expression was analyzed by western blotting using tumors sampled at 24 days after tumor implantation for the control group and at 108 days after administration for the erlotinib-treated group. AXL was markedly upregulated in erlotinib-treated tumors, though expression was not observed in all tumor samples because of the high heterogeneity. AXL phosphorylation was inhibited at 6 h after the last DS-1205b administration (Figure 5D). It was reduced after 21 h of the last administration, however, the antitumor effects were maintained.

### Antitumor effects of DS-1205b in an acquired osimertinib resistance subcutaneous xenograft model

**Figure 6:**
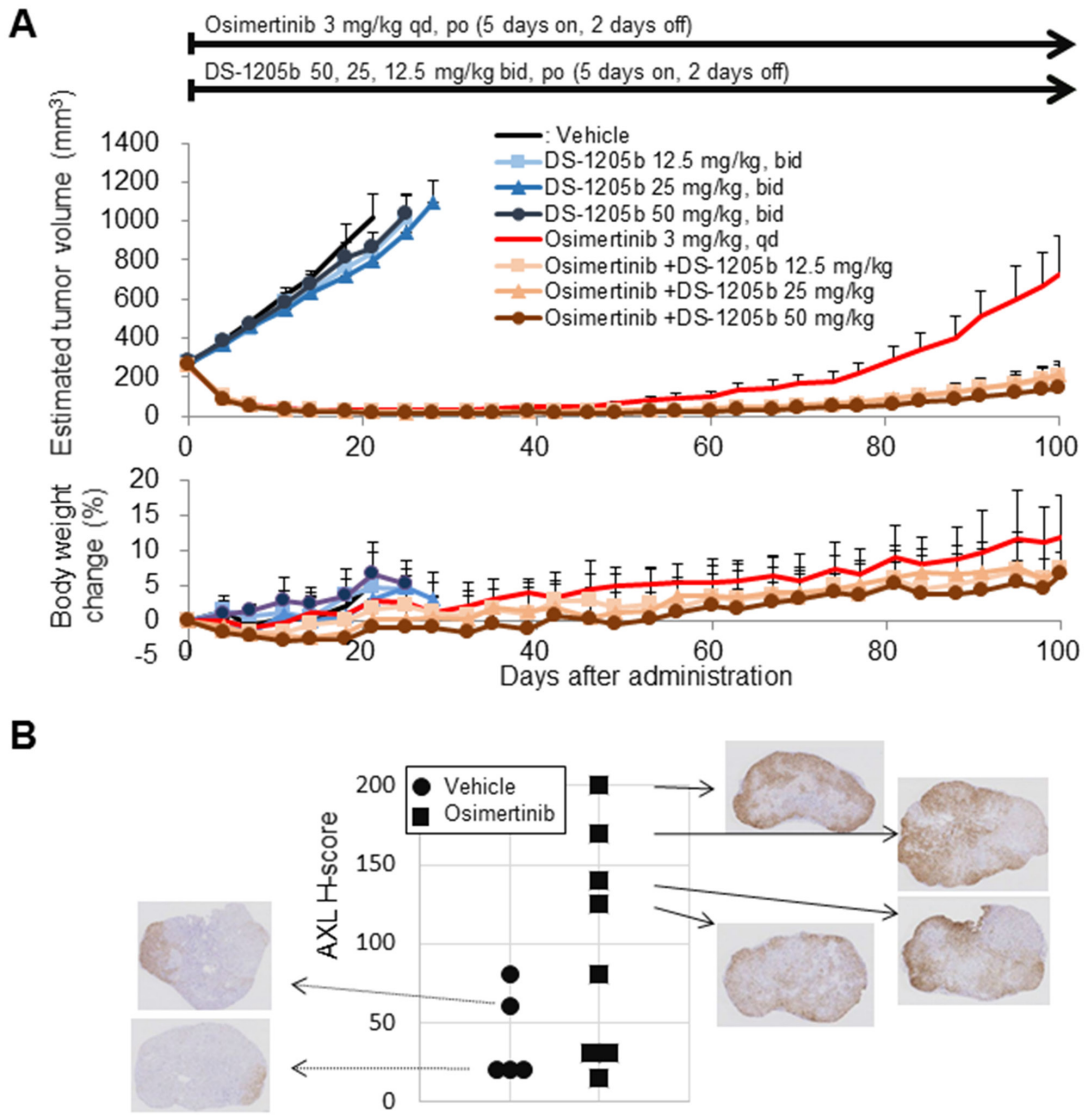
DS-1205b delays osimertinib-acquired resistance in an HCC827 sc xenograft model, and the underlying mechanism. **(A)** Addition of DS-1205b to osimertinib delays the onset of drug resistance in a human EGFR-mutant NSCLC xenograft model. Mice with subcutaneous xenograft human HCC827 tumors were treated as indicated. For vehicle and DS-1205b monotherapy groups, data are the mean ± SE (*N* = 5); for osimertinib monotherapy and the combination groups, data are the mean ± SE (*N* = 8), and body weight change data are the mean ± SD. **(B)** AXL immunostaining images and H scores for HCC827 xenograft tumors are shown (sampling; day 100). AXL immunostaining images and AXL signal intensities and H scores (see Materials and Methods) for HCC827 xenograft tumors are shown.

Using the same mouse model, delay of HCC827 tumor growth by DS-1205b upon acquired resistance to osimertinib was evaluated. On day 100, the mean tumor volume in the group administered osimertinib monotherapy was 727.5 mm^3^. The mean tumor volumes of the groups administered osimertinib in combination with DS-1205b at 12.5, 25, or 50 mg/kg were 205.1, 211.3, and 145.8 mm^3^, respectively. In all groups receiving osimertinib in combination with DS-1205b, tumor volumes were lower than those in the osimertinib monotherapy group. DS-1205b significantly delayed the tumor volume increase and days to reach initial tumor volume in a dose-dependent manner at doses of 12.5–50 mg/kg in the osimertinib acquired resistance model (Figure 6A).

Immunostaining for AXL expression and H scores for HCC827 xenograft tumors are shown in Figure 6B (tumors were sampled on day 100). The osimertinib-treated groups had higher H scores for AXL expression than the vehicle control group (mean ± SD for H score, 40.0 ± 28.3 for vehicle control; 98.8 ± 70.2 for osimertinib monotherapy), suggesting that enhanced AXL expression may contribute to acquired resistance to osimertinib, at least in this model. AXL expression was heterogeneously distributed in the tumors.

**Figure 7:**
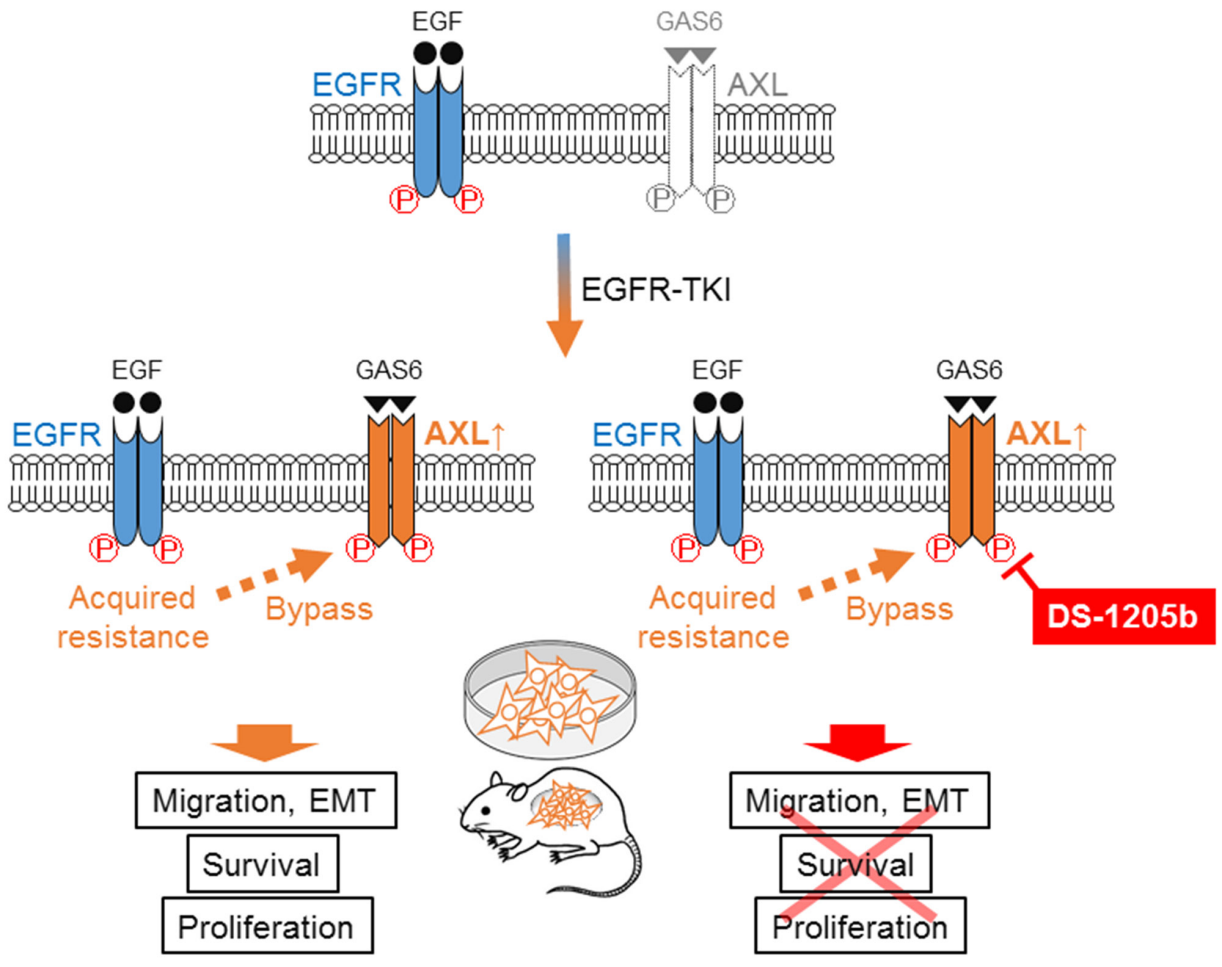
Scheme of mechanism of DS-1205b inhibition. Schematic diagram showing the mechanism of EGFR-TKI acquired resistance because of AXL activation in NSCLC cells harboring EGFR mutations. AXL upregulation works as a bypass signal of EGFR-TKI acquired resistance in EGFR-mutated NSCLC. DS-1205b inhibits AXL phosphorylation, thus delaying drug resistance or inducing combination effects with EGFR-TKI.

Our results indicated that AXL upregulation works, at least in part, as a bypass signal of EGFR-TKI acquired resistance in NSCLC cells harboring EGFR mutations. DS-1205b inhibited AXL phosphorylation and consequently led to a delay in drug resistance or combination effects with EGFR TKIs (Figure 7).

## DISCUSSION

This study showed that DS-1205b delayed the onset of resistance and overcame acquired resistance to EGFR TKIs in a human EGFR-mutated NSCLC (T790M-negative) xenograft model. In clinic, EGFR TKIs significantly improve the overall survival rates of patients with advanced EGFR-mutant NSCLC. However, despite the high rates of initial response to targeted therapy, patients uniformly develop treatment resistance. Accordingly, TKI resistance has become a major therapeutic challenge in the care of patients with EGFR mutations.

There are several mechanisms of resistance to EGFR TKIs [36], and T790M as a gatekeeper mutation is the most frequent cause of acquired resistance to first-generation EGFR TKIs [37, 38]. While osimertinib conquered resistance induced by this mutation, the C797S mutation, which induces resistance to this drug, has emerged [38, 39]. In addition to acquired mutations in EGFR, several other mechanisms, such as bypass mechanisms in EGFR-mediated signaling [40], epithelial-mesenchymal transition (EMT) [32, 41, 42], pathological transformation to small cell lung cancer [43], immune escape [44], and downstream activation of the RAS or PI3K pathways [31] have been reported. Induced AXL expression may be one of the bypass mechanisms. In the current study, it was demonstrated that after treatment with erlotinib or osimertinib, AXL expression was increased in the HCC827 xenograft. Namba et al. recently reported AXL upregulation in a xenograft model upon long-term osimertinib treatment [45]; however, there is currently no evidence of the involvement of AXL upregulation in acquired resistance *in vivo*. When erlotinib was administered in combination with DS-1205b, its activity was restored, suggesting that AXL overexpression indeed contributes to resistance to EGFR TKIs. Byers et al. developed a robust EMT signature that predicts resistance to EGFR and PI3K/AKT inhibitors, highlighted different patterns of drug responsiveness in epithelial and mesenchymal cells, and identified AXL as a potential therapeutic target for overcoming EGFR TKI resistance associated with the mesenchymal phenotype [32, 42]. The current study showed that DS-1205b in combination with osimertinib more effectively inhibited phosphorylation than osimertinib monotreatment, and AKT phosphorylation was completely inhibited, even at the lowest dose (16 nM), under cotreatment. Thus, combination treatment with DS-1205 might prolong the therapeutic benefit of this EGFR-TKI by inhibiting the bypass pathway.

As AXL has numerous physiological functions, it is important to know its exact biological functions and to evaluate the most beneficial use of inhibitors. Recently, Mak et al. developed a patient-derived, pan-cancer EMT signature using 11 distinct tumor types from The Cancer Genome Atlas [46]. Mesenchymal tumors showed similar patterns of gene, protein, and miRNA expression, regardless of the cancer type. Tumors with mesenchymal EMT scores not only had higher AXL expression, but also expressed high levels of multiple immune checkpoints, including PD1, PD-L1, PD-L2, CTLA4, OX40L, CXCR4, CXCR6, and CXCR16 [47]. This finding, which was validated in an independent patient cohort, highlights the potential for utilizing EMT status, regardless of cancer type, as an additional selection tool to select patients who may benefit from immune checkpoint blockade. With regard to the role of AXL in immune evasion, emerging data show that increased AXL expression is involved in the anti-PD-1 or PD-L1 resistance program in non-responders [48–50]. Therefore, it is important to further clarify the biological roles of AXL in the context of anti-PD-1 or PD-L1 therapy, to develop novel approaches in cancer therapy.

We generated an AXL-overexpressing cell line, NIH3T3-AXL [4, 51], as an artificial model of tumorigenesis driven by AXL, and we used it to evaluate the activities of DS-1205b *in vitro* and *in vivo*. There are several lines of evidence that AXL is involved in tumor metastatic processes, such as cell migration and invasion [13, 52], and we confirmed that DS-1205b inhibited hGAS6-dependent cell migration *in vitro.* In the NIH3T3-AXL xenograft model, DS-1205b showed a potent tumor growth-inhibitory effect *in vivo.* This result strongly suggested that DS-1205b clearly inhibits AXL function *in vivo* when it is systemically administered. Several AXL inhibitors are under development, including selective and multi-targeted kinase inhibitors [19, 53–55]. It should be noted that multi-kinase inhibitors might have insufficient AXL inhibitory activity because of their broad spectrum or might demonstrate off-target toxicity. In this regard, a selective AXL inhibitor would be more convenient and efficacious in mono- or combination therapy. Bemcentinib, also known as BGB324 or R428, is an experimental oral small-molecule AXL kinase inhibitor [35]. Bemcentinib was licensed from Rigel Pharmaceuticals by BerGenBio and is currently under investigation in phase II trials in various solid and hematological tumors as monotherapy and in combination with immunotherapy, chemotherapy, and targeted therapeutics as a forefront runner in development (ClinicalTrials.gov IDs: NSCLC; NCT03184571, NCT02424617, triple negative breast cancer; NCT03184558, metastatic melanoma; NCT02872259, and acute myelogenous leukemia or myelodysplastic syndromes; NCT02488408). Recently, BGB324 in addition to its ability to inhibit AXL has been reported [56]. However, the molecular mechanisms of BGB324 in regulating cancer cell growth and metastasis have not been thoroughly investigated. It has been reported that BGB324 induced cancer cell apoptosis [56–58], but the role of AXL inhibition in BGB324-induced apoptosis has not been clarified. We evaluated the potency of BGB324 to inhibit hGAS6-dependent NIH3T3-AXL cell migration, AXL phosphorylation, and cell growth in comparison with DS-1205b. The EC_50_ of BGB324 was 132.3 nM, which was a 49-fold lower activity based on the area under concentration curve (AUC) than that of DS-1205b. Furthermore, its AXL phosphorylation-inhibitory activity was less potent. However, the lower GI_50_ and the apoptosis phenotype was observed at a similar concentration, consistent with a recent report [58]. In terms of AXL inhibition, DS-1205b can be thus expected to show better efficacy than BGB324 in future clinical studies. As DS-1205b is a potent and highly selective AXL inhibitor, the outcome of clinical trials of DS-1205b is highly anticipated. A phase I study in combination with osimertinib is underway (ClinicalTrials.gov IDs: NCT03255083, NCT03599518).

## MATERIALS AND METHODS

### Test compounds

DS-1205b (chemical name: *N*-[4-(2-amino-5-{4-[(2*R*)-1,4-dioxan-2-ylmethoxy]-3-methoxyphenyl}pyridin-3-yl)-3-fluorophenyl]-5-methyl-4′-oxo-1′-(tetrahydro-2*H*-pyran-4-ylmethyl)-1′,4′-dihydro-2,3′-bipyridine-5′-carboxamide 1 4/5 sulfate trihydrate, molecular formula: C_41_H_42_FN_5_O_7_·1 4/5H_2_SO_4_·3H_2_O) was synthesized by Daiichi Sankyo Co., Ltd. (Tokyo, Japan). The molecular formula was redefined as a non-stoichiometric sulfate hydrate, DS-1205c, C_41_H_42_FN_5_O_7_·xH_2_SO_4_·yH_2_O (where x is approximately 1 4/5 and y is approximately 3) for clinical trials. Erlotinib and osimertinib were provided by LC Laboratories (Woburn, MA, USA) and Shanghai Sun-shine Chemical Technology Corporation Ltd. (Shanghai, China), respectively. BGB324 was synthesized by Daiichi Sankyo Co., Ltd. (Tokyo, Japan).

### Kinase inhibition assay

AXL, MER, MET, and TRKA kinase inhibition by DS-1205b was determined using the LabChip EZ Reader (PerkinElmer Inc., Waltham, MA, USA) in the presence of 1 mM ATP. DS-1205b dimethyl sulfoxide (DMSO) solution was added to kinase solution and the mixture was incubated at room temperature for 20 min. After the addition of ATP, the reaction plates were incubated at 28°C for 45 min. The kinase panel was evaluated by mobility shift assay (Carna Biosciences, Inc., Natick, MA, USA). The IC_50_ was determined.

### *In vitro* growth inhibition assay

NIH3T3 mouse embryonic fibroblast cells were purchased from CLS Cell Lines Service GmbH (Eppelheim, Germany). Full-length AXL cDNA was inserted into a pLXSN retroviral vector (Clontech Laboratories, Inc., Mountain View, CA, USA) and the construct was transfected into NIH3T3 cells (NIH3T3-AXL). The cells were plated in 96-well plates (Corning, New York, NY, USA) and allowed to attach for 24 h. Then, the cells were treated with DS-1205b or BGB324 at concentrations in the range 1.52–10,000 nM in DMEM with 0.2% DMSO and incubated under 5% CO_2_ at 37°C for 3 days. Growth inhibition was determined by an ATP assay using the CellTiter-Glo^®^ 2.0 Assay (Promega, Madison, WI, USA). The data were analyzed using GraphPad Prism 6.0 (GraphPad Software, Inc., San Diego, CA, USA) to derive a dose-response curve and the GI_50_ values. Each treatment was tested in triplicate at least three times independently, and the results represent the average of the independent experiments.

### Cell morphology evaluation

NIH3T3-AXL cells were plated at 1 × 10^5^ cells/well in 6-well plates and were incubated overnight (5% CO_2_, 37°C). DMSO, DS-1205b, or BGB324 were added to the culture medium at a final concentration 1 μM (for DS-1205b and BGB324 treatments) for 24 h. Phase-contrast images were captured with Leica DMI6000B microscope, using a Leica N PLAN L 20×/0.35 objective lens.

### Migration inhibition assay

Inhibition of NIH3T3-AXL cell migration by DS-1205b or BGB324 was evaluated using a real-time cell analyzer (RTCA) DP instrument (xCELLigence; Roche Diagnostics, Basel, Switzerland), and the CI, a dimensionless parameter, was calculated as a relative change in measured electrical impedance to represent cell migration. DS-1205b or BGB324 was added to the lower chamber of the RTCA well, together with hGAS6 as the chemoattractant. The cell migration kinetics across the membrane separating the chambers of the RTCA well were continuously recorded by the RTCA DP instrument for 12 h in an incubator set at 37°C, with 5% CO_2_. The CI AUC_0–12_ indicates the sum of each CI for 12 h, and the EC_50_ was calculated based on the CI AUC_0–12_.

### *In vivo* growth inhibition assay

NIH3T3-AXL tumor blocks (2 mm × 3 mm) were implanted in the right flanks of female NOD/Shi-scid IL-2Rγ KO Jic mice. Eighteen days after implantation, the mice were orally administered vehicle (0.5% methylcellulose) or DS-1205b at 50, 25, 13, 6.3, or 3.1 mg/kg bid for 5 days. The long and short tumor diameters and body weight were assessed at 3–4- and 2–4-day intervals, respectively. Tumor dimensions were measured with a digital caliper and tumor volumes were calculated as long diameter × short diameter^2^/2. Mice were sacrificed before the growing tumors reached 2,000 mm^3^, or when the once-shrunken tumors regrew to the size on day 0. All animal experimental procedures conformed to the guiding principles of the Daiichi Sankyo Co., Ltd. Institutional Animal Care and Use Committee.

### Evaluation of the delaying effect of DS-1205b on acquired erlotinib or osimertinib resistance in a xenograft model *in vivo*

HCC827 cells were purchased from the American Type Culture Collection (Manassas, VA, USA). The cells were inoculated subcutaneously into 6-week-old female CAnN.Cg-Foxn1nu/CrlCrlj mice. Erlotinib (25 mg/kg), osimertinib (3 mg/kg), or 0.5 w/v% methyl cellulose 400 solution were administered orally once per day, and DS-1205b (50, 25, or 12.5 mg/kg bid) was administered orally in accordance with a 5 days on/2 days off schedule. In resistance delay studies, erlotinib and osimertinib were started from day 56 and 45 after tumor inoculation, respectively. Vehicle administration and DS-1205b monotherapy were continued until the tumor volume reached 1,000 mm^3^. Erlotinib and osimertinib monotherapy and the combination with DS-1205b were continued until day 100. Body weight and tumor diameters were measured twice per week.

### Evaluation of the effect of DS-1205b on acquired erlotinib resistance in a xenograft model *in vivo*

Erlotinib-resistant tumors were generated in female CAnN.Cg-Foxn1nu/CrlCrlj mice by subcutaneous inguinal inoculation of HCC827 human NSCLC cells, followed by once-daily erlotinib administration (25 mg/kg) for approximately 9 weeks with a 5 days on/2 days off schedule. After the generation of erlotinib-resistant tumors in the mice, DS-1205b was administered twice per day at a dose of 25 or 50 mg/kg for approximately 7 weeks in combination with erlotinib, which was administered once per day. Tumor length and width and body weight were measured every 3 or 4 days. Tumor volumes on the day of the last administration of erlotinib and DS-1205b were used to evaluate antitumor effects indicated as total growth inhibition by net (TGIn)%. The measurements were continued until the mean tumor volume of the vehicle-treated group reached a volume not exceeding 1,500 mm^3^.

### Western blot analysis of drug-treated cells and tissue samples

Polyvinylidene difluoride membranes containing electrophoretically separated proteins from whole-cell lysates and tumor tissues were probed with antibodies. All antibodies, except anti-actin and rabbit IgG antibodies and Can Get Signal Immunoreaction Enhancer Solution 1 (Can Get Solution 1; Toyobo Co., Ltd., Osaka, Japan), were mixed at 1:1,000. Anti-actin antibody and Can Get Solution 1 were mixed at 1:2,000. For the secondary antibody, anti-rabbit IgG antibody and Can Get Signal Immunoreaction Enhancer Solution 2 (Can Get Solution 2; Toyobo Co., Ltd.) were mixed at 1:2,000. All antibodies were stored at –20°C. The following antibodies were used: anti-AXL (C44G1) rabbit monoclonal antibody (Cell Signaling Technology, Inc., Danvers, MA, USA, #4566), anti-phospho AXL (Tyr702) (D12B2) rabbit monoclonal antibody (Cell Signaling Technology, Inc., #5724), anti-EGFR rabbit polyclonal antibody (Cell Signaling Technology, Inc., #2232), anti-phospho EGFR (Tyr1173) (53A5) rabbit monoclonal antibody (Cell Signaling Technology, Inc., #4407), anti-AKT rabbit polyclonal antibody (Cell Signaling Technology, Inc., #9272), anti-phospho AKT (Ser473) (D9E) rabbit monoclonal antibody (Cell Signaling Technology, Inc., #4060), anti-p44/42 mitogen-activated protein kinase (MAPK) (ERK1/2) rabbit polyclonal antibody (Cell Signaling Technology, Inc., #9102), anti-phospho 44/42 MAPK (ERK1/2) (Thr202/Tyr204) (D13.14.4E) rabbit monoclonal antibody (Cell Signaling Technology, Inc., #4370), anti-β-actin (13E5) rabbit monoclonal antibody (Cell Signaling Technology, Inc., #4970), anti-rabbit IgG, and HRP-linked goat polyclonal antibody (Cell Signaling Technology, Inc., #7074).

### Immunohistochemical analysis of tumor samples

Primary AXL antibody (C89E7, rabbit mAb; Cell Signaling Technology, #8661) was diluted to a final concentration of 3.72 μg/mL with Dako antibody diluent (DAKO REAL Antibody Diluent K3468; Dako Denmark A/S, Glostrup, Denmark). Approximately 4-μm-thick sections were deparaffinized and heat-pretreated with Target Retrieval Solution (pH 9) (Dako Denmark A/S) in a microwave oven for antigen retrieval. Subsequently, the sections were subjected to endogenous peroxidase blocking and protein blocking. The sections were sequentially incubated with the primary antibody for 60 min, EnVision + System-HRP Labelled Polymer Anti-Rabbit (Dako Denmark A/S) as a secondary antibody for 30 min, and Liquid DAB + Substrate Chromogen System (Dako Denmark A/S) for 5 min. The sections were washed after each step. Finally, the sections were counterstained with hematoxylin for 5 min and mounted.

AXL signal intensities were scored on the whole tumor area (excluding necrotic and stromal areas) according to a semi-quantitative grading system, in which the staining intensity was graded as 0 (no staining), 1+ (faint to weak), 2+ (moderate), or 3+ (strong). Representative images for each grade are shown in Figures 3D and 4B. The AXL-stained sections were observed under a BX51 microscope (Olympus Corporation, Tokyo, Japan). The AXL-positive staining area (percent positive) was calculated as the sum of percentages for each score. H scores were calculated in Microsoft Excel 2010 according to the following equation: H score = (3 × % of cells with strong staining) + (2 × % of cells with moderate staining) + (1 × % of cells with weak staining). Group means and SDs were also calculated with regard to percent positive and H scores for each group.

### Statistical analysis

Data are presented as the mean ± SD or mean ± standard error (Excel 2013, Microsoft Corporation, Redmond, WA, USA). Treatment groups were compared with controls by parametric Dunnett’s tests. Spearman’s rank correlation coefficient testing was used to analyze dose-dependent effects. *P-*values <0.05 were considered statistically significant. EC_50_ was calculated using a sigmoid E_max_ model [59, 60]. SAS system release 9.2 was used for statistical analyses and to generate the E_max_ model (SAS Institute, Inc., Cary, NC, USA).

## SUPPLEMENTARY MATERIALS TABLE AND FIGURES



## Abbreviations

AKT: AKT serine/threonine kinase
ATP: adenosine triphosphate
AUC0-t: area under the plasma concentration-time curve to time t
AXL: AXL receptor tyrosine kinase
bid: twice daily
CI: cell index
DMSO: dimethyl sulfoxide
EGFR: epidermal growth factor receptor
EMT: epithelial-mesenchymal transition
ERK: extracellular signal-regulated kinase
hGAS6: human growth arrest-specific gene 6
MAPK: mitogen-activated protein kinase
NSCLC: non-small cell lung cancer
qd: once a day
TAM: TYRO-3, AXL, and MER
TKI: tyrosine kinase inhibitor
