# Supplementary material for: DS-1205b, a novel selective inhibitor of AXL kinase, blocks resistance to EGFR-tyrosine kinase inhibitors in a non-small cell lung cancer xenograft model
Source: Oncotarget. 2019 Aug 27;10(50):5152–67. doi: 10.18632/oncotarget.27114 (PMC6718264; doi:10.18632/oncotarget.27114)
Supplement: Supplementary file 1 [file oncotarget-10-5152-s001.pdf]

DS-1205b, a novel selective inhibitor of AXL kinase, blocks resistance to EGFR-tyrosine kinase inhibitors in a non-small cell lung cancer xenograft model

SUPPLEMENTARY MATERIALS

Supplementary Methods

General protocol for the synthesis of DS-1205b

All solvents and reagents were used as acquired from commercial sources, without purification. Silica gel or amino silica gel column chromatography was performed on commercially available column cartridges and a Biotage SP1, a Yamazen YFLC-W-Prep2XY, or a Shoko scientific Purif-espoir2 purification system. Thin-layer chromatography (TLC) was performed on Merck TLC plates precoated with silica gel 60 F_{254}, 60 NH_2 F_{254}s, and Wako TLC plates precoated with NH_2 silica gel NH_2 F_{254}. ^{1}H-NMR spectra were recorded on a JEOL JNM-ECX400P and JNM-ECS400 spectrometers. ^{1}H-NMR chemical shifts are given in ppm (δ) from tetramethylsilane, which was used as the internal standard. Mass spectra were measured using an Agilent G6130B (APCI) spectrometer.

5-Methyl-4’-oxo-1’-(tetrahydro-2*H*-pyran-4-ylmethyl)-1’,4’-dihydro-2,3’-bipyridine-5’-carboxylic acid

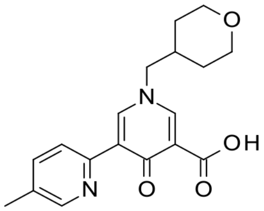

To a solution of (5-methylpyridin-2-yl)acetic acid (0.500 g, 3.31 mmol) in tetrahydrofuran (15.0 mL), 1,1’-carbonylbis-1*H*-imidazole (0.805 g, 4.96 mmol) was added, and the mixture was stirred at room temperature for 2.5 h. In another reaction vessel, monoethyl potassium malonate (1.69 g, 9.92 mmol) and magnesium chloride (0.945 g, 9.92 mmol) were suspended in tetrahydrofuran (25 mL). To the suspension, triethylamine (2.38 mL, 1.74 g, 17.2 mmol) was added under ice cooling. The mixture was stirred at room temperature for 2.5 h and then ice-cooled again. The activated ester solution prepared as mentioned above was added, and the mixture was stirred overnight at room temperature. To the reaction mixture, a 10% aqueous citric acid solution was added, followed by extraction with ethyl acetate five times. The organic layer was washed with a saturated aqueous solution of sodium bicarbonate and brine, and then dried over anhydrous sodium sulfate. After filtration and concentration under reduced pressure, the residue was purified by silica gel column chromatography (n-hexane/ethyl acetate = 57/43–36/64) to yield ethyl 4-(5-methylpyridin-2-yl)-3-oxobutanoate as an oil. This substance was dissolved in toluene (7.5 mL). To the solution, *N*,*N*-dimethylformamide dimethyl acetal (1.89 mL, 14.2 mmol) was added, and the mixture was heated under stirring at 100°C for 3.5 h. The reaction mixture was cooled to room temperature and concentrated under reduced pressure. The residue was dissolved in ethanol (15 mL). To the solution, tetrahydro-2*H*-pyran-4-ylmethanamine (0.520 mL, 4.27 mmol) was added, and the mixture was heated under stirring at 60°C for 3 h. The reaction mixture was cooled to room temperature and concentrated under reduced pressure. The residue was purified by aminosilica gel column chromatography (n-hexane/ethyl acetate = 50/50–0/100, then ethyl acetate/MeOH = 100/0–95/5) to yield a yellow solid. This substance was washed with diethylether and collected to yield ethyl 5-methyl-4’-oxo-1’-(tetrahydro-2*H*-pyran-4-ylmethyl)-1’,4’-dihydro-2,3’-bipyridine-5’-carboxylate as pale yellow solid. This substance was suspended in tetrahydrofuran (10 mL). To the suspension, methanol (5 mL) and a 1 N aqueous sodium hydroxide solution (4.80 mL, 4.80 mmol) were added, and the mixture was stirred at room temperature for 4 h. The reaction mixture was concentrated under reduced pressure. To the residue, 10% aqueous solution of citric acid was added. The precipitated solid was collected to yield the compound [0.466 g, yield: 44% from (5-methylpyridin-2-yl)acetic acid] as a colorless solid. ^{1}H-NMR (DMSO-d_6) δ: 8.86 (1H, d, *J* = 2.4 Hz), 8.80 (1H, d, *J* = 2.4 Hz), 8.53 (1H, s), 8.38 (1H, d, *J* = 8.5 Hz), 7.72 (1H, dd, *J* = 8.5, 2.1 Hz), 4.24 (2H, d, *J* = 7.3 Hz), 3.84 (2H, dd, *J* = 11.2, 3.3 Hz), 3.24 (2H, t, *J* = 11.2 Hz), 2.35 (3H, s), 2.11–2.01 (1H, m), 1.45–1.42 (2H, m), 1.35–1.24 (2H, m). MS (APCI) m/z: 329 $(M+H)^+$.

(2*R*)-2-{[2-methoxy-4-(4,4,5,5-tetramethyl-1,3,2-dioxaborolan-2-yl)phenoxy]methyl}-1,4-dioxane

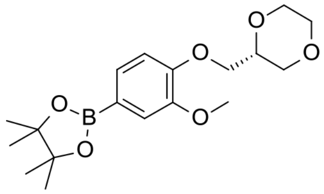

2-Methoxy-4-(4,4,5,5-tetramethyl-1,3,2-dioxaborolan-2-yl)phenol (70.0 g, 280 mmol), (2*R*)-1,4-dioxan-2-ylmethyl methanesulfonate (54.9 g, 280 mmol), and potassium carbonate (77.4 g, 560 mmol) were suspended in *N*,*N*-dimethylformamide (1.40 L), and the suspension was stirred at 95°C for 14 h. After cooling, the insoluble matter was removed by filtration and the solvent was evaporated under reduced pressure. The residue was purified by silica gel column chromatography (n-hexane/ethyl acetate = 2/1) to yield the title compound (81.6 g, yield: 83%) as a colorless solid. ^{1}H-NMR ($CDCl_3$) δ: 7.38 (1H, dd, *J* = 8.0, 1.5 Hz), 7.28 (1H, d, *J* = 1.5 Hz), 6.89 (1H, d, *J* = 8.0 Hz), 4.10–3.93 (4H, m), 3.89 (3H, s), 3.87–3.61 (4H, m), 3.57–3.49 (1H, m), 1.34 (12H, s). MS (APCI) m/z: 351 $(M+H)^+$.

3-Bromo-5-{4-[(2*R*)-1,4-dioxan-2-ylmethoxy]-3-methoxyphenyl}pyridin-2-amine

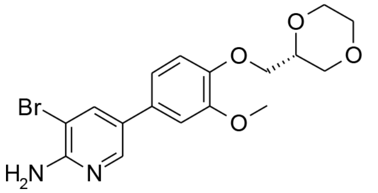

(2*R*)-2-{[2-methoxy-4-(4,4,5,5-tetramethyl-1,3,2-dioxaborolan-2-yl)phenoxy]methyl}-1,4-dioxane (3.50 g, 10.0 mmol), 3-bromo-5-iodopyridin-2-amine (3.00 g, 10.0 mmol), tetrakis(triphenylphosphine)palladium (580 mg, 0.500 mmol), and sodium carbonate (2.10 g, 20.0 mmol) were suspended in dioxane (50 mL) and water (10 mL), and the suspended mixture was stirred at 90°C for 8 h. After cooling, dichloromethane and water were added to the mixture. The organic layer was dried over sodium sulfate, and the solvent was evaporated under reduced pressure. The residue was purified by silica gel column chromatography (chloroform/ethyl acetate = 59/41–28/72) to yield a colorless solid. The slurry was washed with diisopropylether to yield the compound (2.20 g, yield: 56%) as a colorless solid. ^{1}H-NMR (DMSO-d_6) δ: 8.27 (1H, d, *J* = 2.4 Hz), 8.05 (1H, d, *J* = 2.4 Hz), 7.17 (1H, d, *J* = 2.4 Hz), 7.10 (1H, dd, *J* = 8.5, 2.4 Hz), 6.99 (1H, d, *J* = 8.5 Hz), 6.28 (2H, s), 3.98–3.74 (8H, m), 3.69–3.59 (2H, m), 3.53–3.46 (1H, m), 3.42–3.37 (1H. m). MS (APCI) m/z: 395 $(M+H)^+$.

3-(4-Amino-2-fluorophenyl)-5-{4-[(2*R*)-1,4-dioxan-2-ylmethoxy]-3-methoxyphenyl}pyridin-2-amine

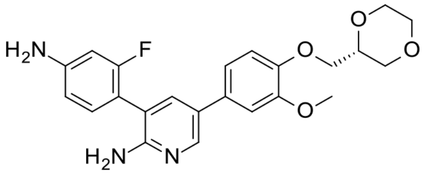

3-Bromo-5-{4-[(2*R*)-1,4-dioxan-2-ylmethoxy]-3-methoxyphenyl}pyridin-2-amine (3.90 g, 9.87 mmol) was dissolved in 1,4-dioxane (33 mL). To the solution, water (6.58 mL), tetrakis(triphenylphosphine)palladium (0.570 g, 0.493 mmol), fluoro-4-(4,4,5,5-tetramethyl-1,3,2-dioxaborolan-2-yl)aniline (2.34 g, 9.87 mmol), and sodium carbonate (2.09 g, 19.7 mmol) were added, and the mixture was stirred at 100°C for 5 h under a nitrogen atmosphere. After cooling, the reaction mixture was separated into organic and aqueous layers by the addition of ethyl acetate and a saturated aqueous ammonium chloride solution. The organic layer was washed with brine and dried over anhydrous sodium sulfate. Then, the solvent was evaporated under reduced pressure. The residue was purified by aminosilica gel column chromatography (n-hexane/ethyl acetate = 60/40–30/70) to yield the compound (2.70 g, yield: 64%) as a colorless foam. ^{1}H-NMR ($CDCl_3$) δ: 8.27 (1H, d, *J* = 2.4 Hz), 7.54 (1H, d, *J* = 2.4 Hz), 7.17 (1H, t, *J* = 8.0 Hz), 7.06–7.01 (2H, m), 6.95 (1H, d, *J* = 8.0 Hz), 6.55 (1H, dd, *J* = 8.0, 2.4 Hz), 6.51 (1H, dd, *J* = 11.5, 2.4 Hz), 4.52 (2H, br s), 4.13–3.52 (12H, m). MS (APCI) m/z: 426 $(M+H)^+$.

N-[4-(2-Amino-5-{4-[(2*R*)-1,4-dioxan-2-ylmethoxy]-3-methoxyphenyl}pyridin-3-yl)-3-fluorophenyl]-5-methyl-4’-oxo-1’-(tetrahydro-2*H*-pyran-4-ylmethyl)-1’,4’-dihydro-2,3’-bipyridine-5’-carboxamide

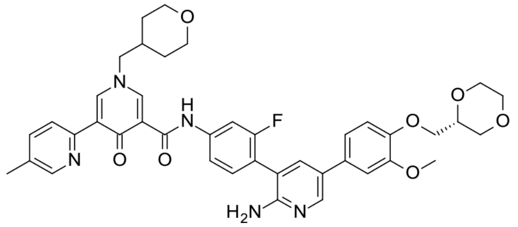

5-Methyl-4’-oxo-1’-(tetrahydro-2*H*-pyran-4-ylmethyl)-1’,4’-dihydro-2,3’-bipyridine-5’-carboxylic acid (17.0 g, 51.7 mmol) was dissolved in *N*,*N*-dimethylformamide (200 mL). After the solution was cooled on ice, *N*-[1-(cyano-2-ethoxy-2-oxoethylideneaminooxy)dimethylamino(morpholino)]uronium hexafluorophosphate (22.1 g, 51.7 mmol) was added, and the mixture was stirred at room temperature for 1 h. After this solution was cooled on ice, a solution of 3-(4-amino-2-fluorophenyl)-5-{4-[(2*R*)-1,4-dioxan-2-ylmethoxy]-3-methoxyphenyl}pyridin-2-amine (20.0 g, 47.0 mmol) in *N*,*N*-dimethylformamide (50 mL) was added, and the mixture was stirred at room temperature overnight. To this mixture, ethyl acetate and saturated aqueous sodium bicarbonate solution were added. The organic layer was washed with brine and dried over anhydrous sodium sulfate, and then concentrated under reduced pressure. The residue was purified by silica gel column chromatography (ethyl acetate/methanol = 100/0–

80/20) to yield the compound (25.0 g, yield: 72%) as a pale yellow solid. ^{1}H-NMR ($CDCl_3$) δ: 13.02 (1H, s), 8.56 (1H, d, J = 2.4 Hz), 8.48–8.46 (2H, m), 8.41 (1H, d, J = 2.4 Hz), 8.31 (1H, d, J = 2.4 Hz), 7.93 (1H, dd, J = 11.8, 2.4 Hz), 7.64–7.59 (2H, m), 7.50 (1H, dd, J = 8.5, 2.4 Hz), 7.38 (1H, t, J = 8.2 Hz), 7.07–7.03 (2H, m), 6.96 (1H, d, J = 8.5 Hz), 4.56 (2H, s), 4.15–3.64 (15H, m), 3.59–3.52 (1H, m), 3.42–3.35 (2H, m), 2.40 (3H, s), 2.22–2.10 (1H, m), 1.62 (2H, d, J = 18.8 Hz), 1.51–1.41 (2H, m). MS (APCI) m/z: 736 $(M+H)^+$.

***N*-[4-(2-Amino-5-{4-[(2*R*)-1,4-dioxan-2-ylmethoxy]-3-methoxyphenyl} pyridin-3-yl)-3-fluorophenyl]-5-methyl-4'-oxo-1'-(tetrahydro-2*H*-pyran-4-ylmethyl)-1',4'-dihydro-2,3'-bipyridine-5'-carboxamide 1 4/5 sulfate trihydrate (DS-1205b)**

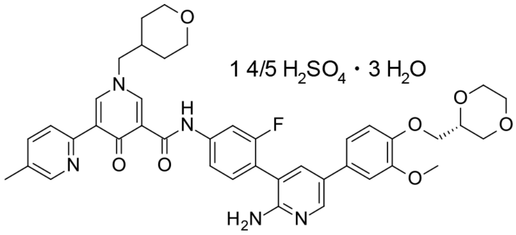

N-[4-(2-Amino-5-{4-[(2*R*)-1,4-dioxan-2-ylmethoxy]-3-methoxyphenyl}pyridin-3-yl)-3-fluorophenyl]-5-methyl-4'-oxo-1'-(tetrahydro-2*H*-pyran-4-ylmethyl)-1',4'-dihydro -2,3'-bipyridine-5'-carboxamide (80.0 g, 109 mmol) was suspended in acetone (1.28 L). To the suspension, water (247 mL) was added and then, 6.00 mol/L sulfuric acid (71.7 mL, 430 mmol) was added dropwise at 40°C. The reaction mixture was stirred at 40°C for 3 days and then suction-filtered with a Buchner funnel, and the precipitated solid was collected by filtration. The solid was washed with a water/acetone mixture (water/acetone = 1/9) to yield the compound (92.3 g, yield: 88%) as a colorless crystalline solid. ^{1}H-NMR (DMSO-d_6) δ: 13.04 (1H, br s), 8.83 (2H, d, J = 9.5 Hz), 8.63 (1H, s), 8.44 (1H, d, J = 8.0 Hz), 8.34 (2H, s), 8.05–7.98 (2H, m), 7.72 (2H, br s), 7.59–7.52 (2H, m), 7.32 (1H, d, J = 2.0 Hz), 7.26 (1H, dd, J = 8.0, 2.0 Hz), 7.07 (1H, d, J = 8.0 Hz), 4.22 (2H, d, J = 7.5 Hz), 4.01–3.93 (2H, m), 3.90–3.74 (8H, m), 3.70–3.59 (2H, m), 3.53–3.45 (1H, m), 3.40 (1H, dd, J = 11.0, 10.0 Hz), 3.27 (2H, t, J = 11.0 Hz), 2.43 (3H, s), 2.16–2.05 (1H, m), 1.51–1.43 (2H, m), 1.39–1.26 (2H, m). MS (APCI) m/z: 736 $(M+H)^+$. Elemental analysis values for $C_{41}H_{42}FN_5O_7 \cdot 1\ 4/5H_2SO_4 \cdot 3H_2O$; calculated: C, 50.96; H, 5.38; N, 7.25; F, 1.97; S, 5.97; found: C, 50.98; H, 5.36; N, 7.23; F, 1.97; S, 6.19.

Supplementary Table 1: Kinase inhibitory activity of DS-1205b toward 161 kinases

Kinase	Inhibition* (%)	Kinase	Inhibition* (%)
**ABL**	<0 / <0	**FAK**	<0 / <0
**ACK**	<0 / <0	**FER**	10.7 / 16.4
**ALK**	<0 / <0	**FES**	3.5 / 5.0
**ARG**	2.2 / 2.3	**FGFR1**	<0 / <0
**AXL**	82.1 / 99.1	**FGFR2**	<0 / 0.9
**BLK**	1.8 / 3.8	**FGFR3**	<0 / 5.0
**BMX**	<0 / <0	**FGFR4**	1.9 / 1.3
**BRK**	3.7 / 43.9	**FGR**	8.4 / 3.2
**BTK**	12.5 / 6.2	**FLT1**	3.8 / 3.2
**CSK**	<0 / <0	**FLT3**	13.4 / 14.9
**DDR1**	1.4 / 2.0	**FLT4**	1.6 / 0.3
**DDR2**	1.9 / 1.5	**FMS**	<0 / 7.4
**EGFR**	2.7 / 2.2	**FRK**	4.0 / 7.6
**EGFR (d746-750)**	2.9 / 1.3	**FYN (isoform a)**	<0 / 1.9
**EGFR (d746-750/T790M)**	2.3 / 3.9	**FYN (isoform b)**	0.8 / 3.8
		**HCK**	4.5 / 4.9
**EGFR (L858R)**	0.7 / 3.3	**HER2**	0.9 / 2.4
**EGFR (L861Q)**	<0 / 2.5	**HER4**	6.7 / 8.7
**EGFR (T790M)**	<0 / 1.2	**IGF1R**	<0 / <0
**EGFR (T790M/L858R)**	2.8 / 5.2	**INSR**	2.5 / 4.7
**EPHA1**	<0 / <0	**IRR**	3.3 / 18.9
**EPHA2**	<0 / <0	**ITK**	0.8 / 0.2
**EPHA3**	<0 / <0	**JAK1**	1.9 / 1.9
**EPHA4**	<0 / <0	**JAK2**	2.9 / 1.6
**EPHA5**	<0 / <0	**JAK3**	<0 / <0
**EPHA6**	<0 / <0	**KDR**	<0 / <0
**EPHA7**	0.2 / 11.7	**KIT**	1.4 / 3.1
**EPHA8**	<0 / <0	**LCK**	4.2 / 8.3
**EPHB1**	<0 / 0.1	**LTK**	3.9 / 8.0
**EPHB2**	<0 / <0	**LYNa**	1.4 / 3.4
**EPHB3**	3.0 / 1.3	**LYNb**	0.4 / 0.9
**EPHB4**	<0 / <0	**MER**	29.5 / 84.9
**Kinase**	**Inhibition* (%)**	**Kinase**	**Inhibition* (%)**
**MET**	7.8 / 51.4	**CaMK4**	0.4 / 0.8
**MUSK**	1.1 / 2.0	**CDC2/CycB1**	2.1 / 0.0
**PDGFRα**	<0 / <0	**CDC7/ASK**	<0 / <0
**PDGFRβ**	<0 / <0	**CDK2/CycA2**	0.9 / <0
**PYK2**	<0 / 3.7	**CDK2/CycE1**	3.9 / <0

(Continued)

**RET**	<0 / <0	**CDK3/CycE1**	2.9 / <0
**RET (G691S)**	<0 / <0	**CDK4/CycD3**	<0 / <0
**RET (M918T)**	<0 / <0	**CDK5/p25**	<0 / <0
**RET (S891A)**	<0 / <0	**CDK6/CycD3**	<0 / 0.4
**RET (Y791F)**	<0 / <0	**CDK7/CycH/MAT1**	5.0 / 0.7
**RON**	<0 / 9.3	**CDK9/CycT1**	<0/ <0
**ROS**	4.0 / 6.4	**CHK1**	<0 / 2.8
**SRC**	4.6 / 5.0	**CHK2**	<0 / <0
**SRM**	5.3 / 5.4	**CK1α**	<0 / <0
**SYK**	<0 / <0	**CK1ε**	0.1 / 1.4
**TEC**	3.3 / 13.0	**CK2α1/β**	1.6 / <0
**TIE2**	2.0 / 14.9	**CLK1**	<0 / 0.7
**TNK1**	1.5 / 0.7	**CLK2**	<0 / 0.3
**TRKA**	6.7 / 50.4	**DAPK1**	8.0 / <0
**TRKB**	1.5 / 21.8	**DYRK1A**	<0 / <0
**TRKC**	4.8 / 34.4	**DYRK1B**	<0 / 1.4
**TXK**	<0 / 2.6	**Erk1**	<0 / <0
**TYK2**	0.8 / <0	**Erk2**	<0 / <0
**TYRO3**	1.0 / 27.5	**Erk5**	0.4 /1.4
**YES**	<0 / <0	**GSK3α**	0.8 / 0.7
**AKT1**	2.3 / <0	**GSK3β**	<0 / <0

Kinase	Inhibition* (%)	Kinase	Inhibition* (%)
**MAPKAPK2**	<0 / <0	**PIM2**	<0 / <0
**MINK**	1.5 / <0	**PKACα**	0.7 / <0
**MST1**	3.4 / 2.3	**PKCα**	6.2 / 0.8
**NEK1**	<0 / <0	**PKCε**	9.9 / 7.7
**NEK2**	1.5 / 0.1	**PKD2**	<0 / <0
**NEK6**	0.7 / 1.9	**PLK1**	<0 / <0
**NEK7**	1.2 / 4.5	**PLK3**	7.3 / 1.7
**NEK9**	0.3 / 3.2	**QIK**	1.1 / <0
**p38α**	<0 / <0	**ROCK1**	3.0 / 2.9
**p38β**	<0 / <0	**RSK1**	1.1 / <0
**p38γ**	<0 / 0.7	**RSK3**	0.1 / <0
**p38δ**	<0 / <0	**RSK4**	17.4 / <0
**p70S6K**	0.8 / 1.6	**SGK**	1.8 / 0.1
**PAK2**	7.4 / <0	**SIK**	<0 / <0
**PBK**	<0 / <0	**skMLCK**	<0 / 0.4
**PDK1**	<0 / 0.8	**TNIK**	0.0 / 3.7
**PIM1**	<0 / <0	**TSSK1**	<0 / 2.5

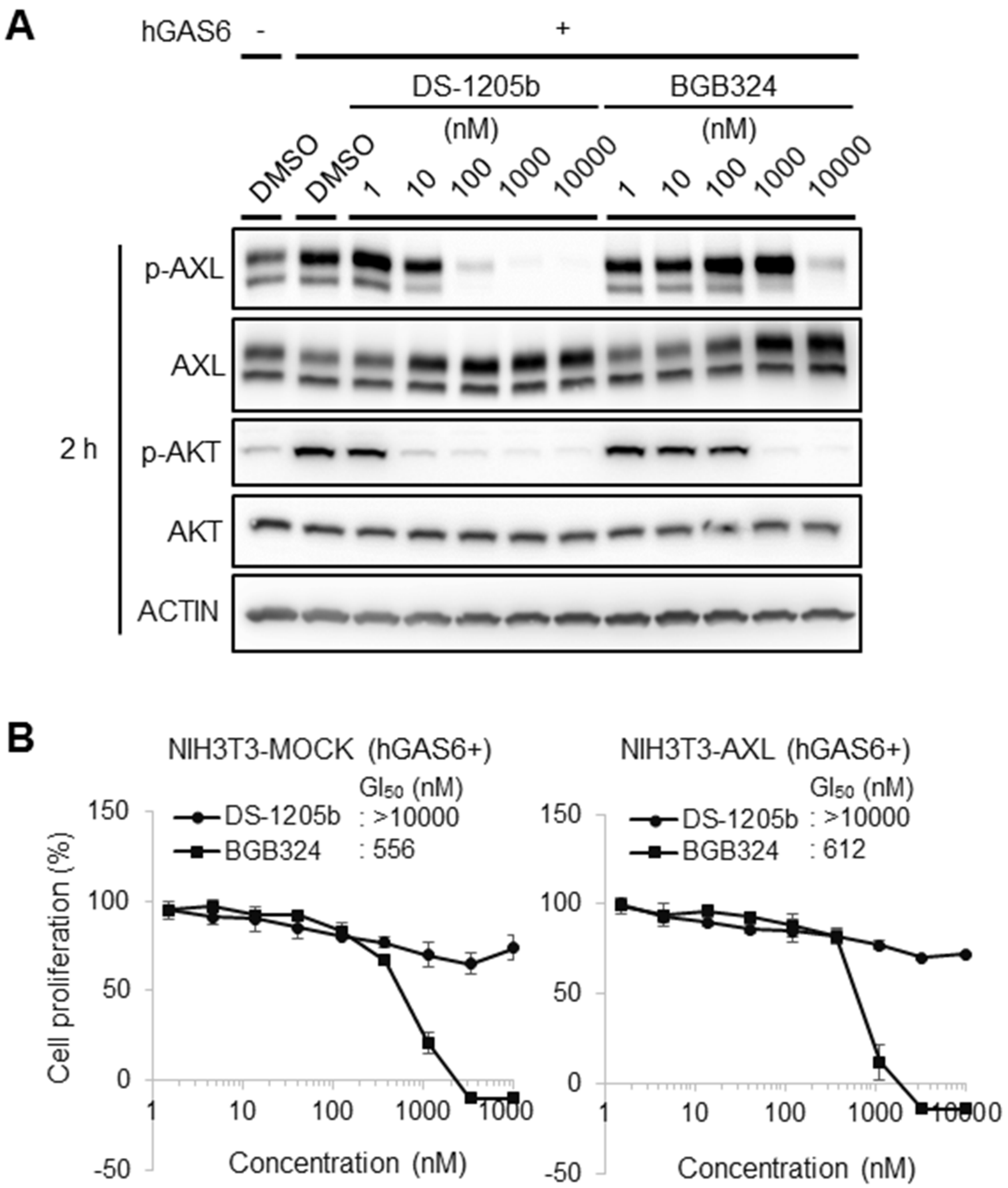

Supplementary Figure 1: Effects of DS-1205b and/or BGB324 in hGAS6 stimulated NIH3T3-AXL cells. **(A)** *In vitro* phosphorylation inhibition assay of AXL and AKT. NIH3T3-AXL cells were treated with DS-1205b or BGB324 for 2 h and then subjected to western blot analysis. The hGAS6 was added to culture medium at 500 ng/mL as final concentration for 10 min before cell harvest. Detailed information on the antibodies used is given in the Materials and Methods. **(B)** Growth inhibitory activities as assessed by ATP assay. The hGAS6 was added to culture medium at 500 ng/mL as final concentration. The graph shows mean inhibition at each concentration point with the SD ($N = 3$).

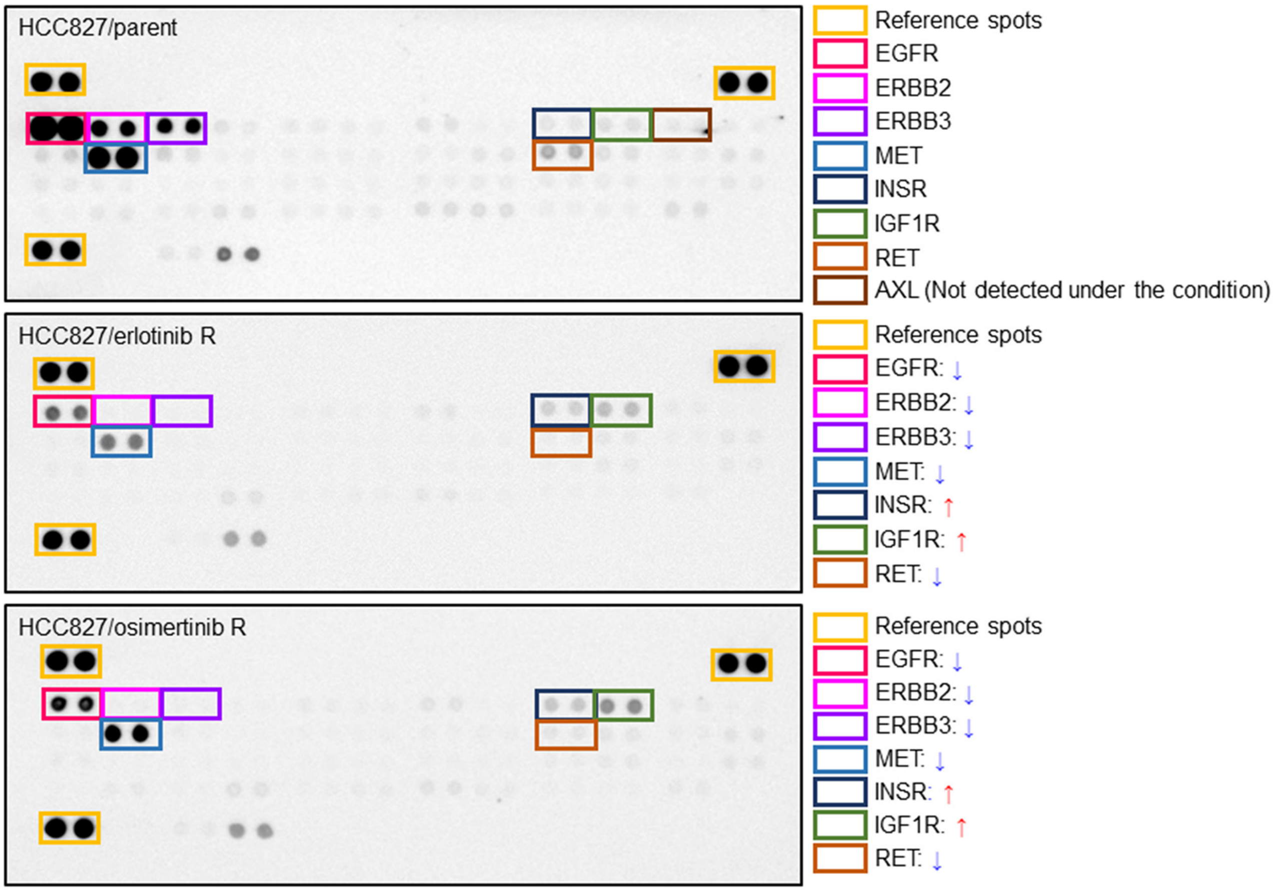

Supplementary Figure 2: Phospho-receptor tyrosine kinase (RTK) array. The relative phosphorylation of 49 different RTKs was measured with Human Phospho-RTK Array Kit (#ARY001B) from R&D systems. Parental, erlotinib resistant, and osimertinib resistant cells were lysed after approximately 72 h culture and assays were carried out according to manufacturer's protocols. The arrows mean more than fivefold changes in detection signals levels vs parental cells.
